# Effect of melatonin on otoprotection in rodents: a systematic review with meta-analysis

**DOI:** 10.1016/j.bjorl.2023.101288

**Published:** 2023-07-04

**Authors:** Natália Lombardi Assumpção, Juliana Gusmão de Araújo, Lucieny Silva Martins Serra, Vanessa Veis Ribeiro, Maria Luiza Queiroz Sampaio, Andressa Alves Caram, André Luiz Lopes Sampaio

**Affiliations:** a Universidade de Brasília, Faculdade de Medicina, Laboratório de Pesquisa em Otorrinolaringologia, Brazil; b Universidade de Brasília, Faculdade de Ceilândia, Departamento de Fonoaudiologia, Brazil

**Keywords:** Melatonin, Rodents, Ototoxic agents, Otoprotection

## Abstract

**Objectives:**

To determinate the otoprotective efficacy of melatonin.in experimental models of rodents through a systematic review of the literature.

**Methods:**

Altogether, 154 articles were found in four databases. The PICOS strategy (Population, Intervention, Comparison, and Outcome) was used to define the eligibility criteria. Studies that met the inclusion criteria for the second step were included in a qualitative synthesis. Each study type was analyzed with the CAMARADES quality of assessment’s checklist and the SYRCLE RoBS risk of bias.

**Results:**

Seven articles were selected, and four were included in the meta-analysis. It was possible to obtain seven outcomes according to the standard auditory frequencies presented among the studies, considering a minimum of three standard frequencies. The outcomes analyzed were for the frequencies of 1500, 2000, 3000, 4000, 5000, 6000, and 8000 Hz.

**Conclusion:**

Melatonin can provide protection against the ototoxic effects of cisplatin and aminoglycosides at 5000 Hz, 6000 Hz, and 8000 Hz, thereby minimizing the reduction in Otoacustic Emissions (OAE) amplitude. The same effect was not observed in the lower frequencies. Despite the limited number of studies that were evaluated, the results appeared consistent in higher frequencies. However, the methodology of the available studies did not meet the necessary methodological rigor that promotes the safe replicability of these studies.

## Highlights

• Melatonin provide protection against the ototoxic effects at 5, 6 and 8 kHz.
• The available paper’s methodology did not meet the rigor for replicability.
• New studies that establish a dose-effect curve of melatonin should be encouraged.

## Introduction

Hearing impairment is characterized by a mild or profound sensorineural or conductive alteration. Data from the World Health Organization (WHO) show that 10% of the world population is affected by this condition.^1^

Regardless of the age at which it is established, deafness can cause impact to the quality of life, work activity, and behavioral changes in an individual. Besides, it may also contribute to the occurrence of dementia.^2^, ^3^ When such a condition is established in the pre-lingual phase, the impact is more significant. It can generate lack of access to oral language, in the absence of an early diagnosis.^4^

Melatonin acts in the elimination of free radicals and has antioxidant properties. It is a hydrophilic and hydrophobic molecule that is released as it is synthesized within the pinealocytes of the pineal gland. This release occurs into the cerebrospinal fluid of the Central Nervous System (CNS) during the night.^5^ The mobilization of the intracellular enzymatic system and the direct chelation of Reactive Oxygen Species (ROS) are mechanisms that participate in the antioxidant capacity of this hormone.^5^

Ototoxicity is a clinical effect caused by certain chemicals that impair the hearing function. It is characterized by a sensorineural hearing loss above 25 dB in one or more frequencies, with or without manifestations of vertigo or imbalance.^6^, ^7^ Aminoglycosides are antibiotics whose ototoxicity is irreversible and those which present variable cochleotoxicity and vestibulotoxicity^8^, ^9^ Chemotherapeutic agents interfere in the mechanisms of cell survival, proliferation, and migration. However, they act in a nonspecific way, damaging malignant and normal cells.

Treatment with antineoplastic drugs can cause ototoxicity.^10^, ^11^, ^12^, ^13^, ^14^ The primary mechanism of cell death induced by these compounds is the generation of Reactive Oxygen Species (ROS) with the induction of apoptosis of the hair cells of the cochlea and vestibule.^15^ It is inferred that antioxidant agents counteract this action by promoting otoprotection.

The most effective method of assessing cochlear ototoxicity is through objective audiological tests such as Evoked Otoacoustic Emissions (OAE).

Currently, no drug can prevent or cure deafness due to ototoxicity. The use of antioxidants, such as melatonin, is a promising attempt to minimize the effects of this condition in humans. Several experiments have been performed in animal models that test melatonin as an otoprotective substance.^16^, ^17^, ^18^, ^19^, ^20^

The main objective of this study was evaluating the efficacy of melatonin as an otoprotective agent, based on experimental models, through a systematic review.

## Methods

The current study is a systematic literature review followed by a meta-analysis of the collected data.

### Research strategy

The search strategy followed the criteria recommended by the Preferred Reporting Items for Systematic Reviews and Meta-Analyses (PRISMA) strategy. The protocol was registered on December 16th, 2023 in the database PROPERO by the number CRD42022287455.

The databases used included Lilacs, Pubmed, Web of Science, Scopus, Embase, and the gray literature from the Google Scholar. Each database used different search strategies for keywords and Medical Subject Headings (MeSH). These terms were combined and customized for each database that was searched using the following keywords in the search strategy: “Rodentia”, “Beaver”, “Ototoxicity”, “Drug-Induced Otological Toxicity”, “Drug Related Cochleotoxicity”, “Intervention” and “Melatonin”.

After the search, the references of each database were exported to the RAYYAN program registering all duplicate articles found in the scientific literature, promote greater reliability in selecting articles, and define the eligibility criteria. The period of this search was from the creation of the platforms until August 31st, 2022, and there was no language restriction.

### Eligibility criteria

PICO strategy (population, intervention, comparison, outcomes)^21^, ^22^ was used to define the eligibility criteria. The selected articles should explicitly include the following information: 1) population: rodent animals exposed to ototoxic agents; 2) intervention: use of melatonin as the sole otoprotective agent; 3) comparison: concomitant use with placebo or any antioxidant; 4) outcome: results of audiological tests such as Evoked Otoacoustic Emissions and Brainstem Auditory Evoked Potential and results of histological evaluations.

The exclusion criteria included: 1) studies that did not use melatonin as the sole otoprotective agent; 2) studies in humans; 3) studies with duplicate data; 4) observational studies; 5) letters to the editor.

Studies were screened for eligibility in the screening phases considering the inclusion and exclusion criteria. In the first phase, all studies were selected by two independent reviewers based on the analysis of titles and abstracts. In the second phase, the same reviewers analyzed the full text of each selected article based on the already established inclusion and exclusion criteria and added the justification for exclusion for each discarded study. In case of conflict, a third reviewer would be consulted.

### Qualitative overview

The instruments used to assess the quality of assessment and the risk of bias were CAMARADES end the SYRCLE RoBS. Items 4, 6 and 7 of the CAMARADES check list were adapted for this review. The items of both instruments were scored as “yes”, “no” or “unclear”.

During this phase, the reviewers independently applied the instruments. There was no disagreement between them, and consultation with the third reviewer was waived.

### Quantitative overview

To perform the meta-analysis, it was necessary to survey the graphic data of each study. The data were not described in the original articles. Thus, it was necessary to send e-mails to the corresponding authors. Only one author responded to the request. For the others, the Graphreader.com tool was used to obtain the data.

The data extracted for the meta-analysis were organized according to the standard hearing frequencies presented in each study. Those that were analyzed by at least three studies were grouped to compose each outcome. Thus, the auditory frequencies evaluated as outcomes were 1500, 2000, 3000, 4000, 5000, 6000, and 8000 Hz.

Numerical data analysis was performed and reported according to Review Manual 5.4 (Cochrane-Collaborating Center, Denmark). The mean difference was used as a measure of effect. A random effects model was fitted to the data. Heterogeneity (tau²) was estimated using the restricted maximum likelihood estimator (Viechtbauer 2005). In addition to the estimation of tau², the Q-test for heterogeneity (Cochrane 1954) and I² statistics were reported. The random effects model was used for the detected outcomes with high heterogeneity (i.e., tau² > 25).

## Results

**Figure 1:**
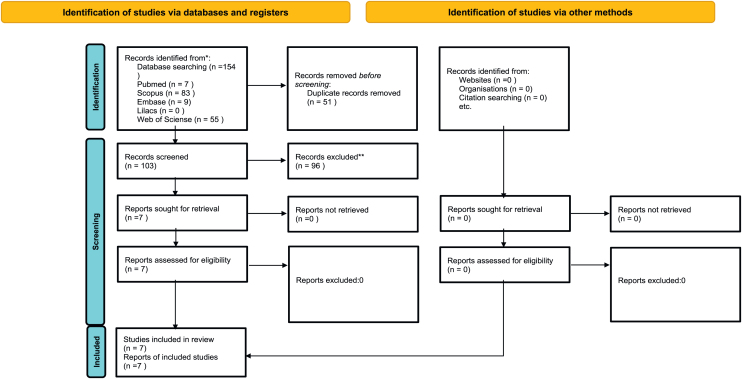
PRISMA flowchart of the article selection process. *Consider, if feasible to do so, reporting the number of records identified from each database or register searched (rather than the total number across all databases/registers). **If automation tools were used, indicate how many records were excluded by a human and how many were excluded by automation tools. From: Page MJ, McKenzie JE, Bossuyt PM, Boutron I, Hoffmann TC, Mulrow CD, et al. The PRISMA 2020 statement: an updated guideline for reporting systematic reviews. BMJ 2021;372:n71. doi: https://doi.org/10.1136/bmj.n71. For more information, visit: http://www.prisma-statement.org/.

A total of 154 articles were identified across four databases. After duplicity screening, 51 articles were excluded, leaving 103 articles. After analysis that included reading the title and abstract, 96 studies were excluded for not meeting the inclusion criteria. Eventually, seven articles were included, as indicated in Fig. 1.

The abstract of the articles included in the qualitative overview can be seen at Table 1.

**Table 1:** Data referring to the articles selected in the systematic review stage.

Author/publication’s year	Sample	Description of experimental groups	Evaluation time and evaluation type	Outcomes
de Araujo et al. ^17^ (2019)	32 Rodents-Rat Wistar/female 3 months old.	4 Groups - Groups — control saline (1 mg/kg): 5 rat/control melatonin (1 mg/kg): rats/cisplatin (10 mg/kg) + melatonin (1 mg/kg): 12 rats/cisplatin (10 mg/kg) + melatonin (1 mg/kg): 10 rats	8 days (D1 and D8) — DPOAE	D1 – (DPOE) no differences between the four groups/D8 – lower values on group cisplatin + saline compared to the saline group at all frequencies. The results did no differ between the saline and melatonin or cisplatin + melatonin groups.
Bas et al.^27^ (2012)	25 Rodents-Rat Wistar/male, age not mentioned	In vivo: groups: S (MP: 200 mL of saline; gelfoam: 40 mL of saline); GM (MP: 10 mg pf GM in 200 mL of saline; gelfoam: 10 mg of GM in 40 mL of saline); GM + DXM; GM + MLT; GM + TCR (MP: 10 mg of GM and DXM, MLT or TCR (MP: 10 mg of GM and DXM, MLT or TCR respectively, with each at 500 mM final concentration in 200 mL of saline; gelfoam: 10 mg of GM and DXM, MLT or TCR, respectively, with each at 500 mM final concentration in 40 mL of saline	In vivo studies: 21 days	In vivo: DPOAE — cochlear treated with saline had values that were similar to the base line values that were obtained before the onset of treatment. Ears treated with GM had a decrease on days 10, 15, 21. No significant differences between basal levels and DPOAE for GM + DXM; GM + MLT and GM + TCR. ABR-S-treated ears only showed a significant elevation in threshold in the gelfoam treatment group at 10 days post-treatment and then a return to a pretreatment threshold at 15 days post-treatment, while cochleae treatment with GM by either gelfoam or via a MP showed significant increases (*p* < 0.05) in ABR threshold with respect to their baseline values on post-treatment days 10–20. In the GM + DXM; GM + MLT and GM + TCR treated groups, the ABR threshold values were similar to those values recorded for the S-treated ears with only a transient elevation in thresholds in the MP GM + DXM, Group at 10 days and in the MP GM + TCR group at 15 days post- treatment. Cytocochleogram. Analysis of the cochleograms constructed from the hair cell counts taken from the organ of Corti FITC phalloidin-stained surface preparations from the five groups of animals indicate a loss of OHCs in the mid- to high-frequency regions of the cochlear treated with GM while the cochleae treated with saline lost very few auditory sensory cells. Treatment of cochleae with GM did not affect IHCs viability in the mid- to high-frequency regions, the treatment of GM-exposed cochleae with either DXM, MLT or TCR prevented most of the GM exposure-induced OHC losses.
Demir et al.^26^ (2015)	24 Rodents-Rat Wistar/male, age not mentioned	3 Groups: control group — single dose of intraperitoneal saline (12 mg/kg) for 5 days and 0.1 cc transtympanic saline for 5 days. Group 2: single dose of intraperitoneal cisplatin (12 mg/kg) and 0.1 cc transtiympanic saline for 5days. Group 3: single dose of intraperitoneal cisplatin (12 mg/kg) and 0.1 mg/mL transtimpanic melatonin for 5 days.	11 days: ABR – realized one day prior to the study and 10 days after the cisplatin dose/EOA – realized on the first day of the study and 10 days after the cisplatin dose.	DPOAE — values and threshold shift at DPOAE were calculated. Group 1 — had significant better thresholds in all frequencies than Group 2. Group 1 and Group 3 did not have any significant differences on acoustic values. Group 3 had significantly smaller decreases at high frequencies than Group 2 ABR values Group 3 had better ABR click at 4000, 60,000 and 8000 Hz; no differences between Group 1 and 3. Pathologic results: no significant differences between the groups. However, when comparing the means, there is less cilia loss and epithelial loss in Group 3 than Group 2
Erdem et al.^19^ (2005)	44 Rodents — Sprague Dawley-Rat com 12 month age	5 Groups all substances intra — penitonially; control group: vehicle: 4 rats; melatonin group (4 mg/kg/day): 4 rats; amikacin group (600 kg/day): 12 rats amikacin + low dose melatonin (0.4 mg/kg/day) group: 12 rats; amikacin + high dose melatonin (4 mg/kg/day) group: 12 rats	DPOAE measurements: day 0, day 5, day 10, day 15	Groups control and melatonin ‒ not significant changes from DPOA. amikacin group ‒ DP GRAM not affected during the study. Amikacin ototoxicity findings in I/O function detected on the 10 days. No differences between day 10 and 15 Group AML: no statistically significant differences in DP-GRAM in the whole study Group AMH: the DP gram and I/O functions amplitude were reduced, and I/O thresholds were increased by means of high dose melatonin administration of amikacin
Lopez-Gonzalez et al.^23^ (2000)	180 Rodents — Wistar Female Rat two months old 20 in each group	9 Groups - Group 1: control; Group 2: gentamicin (200 mg/kg); Group 3: tobramycin (160 mg/kg; Group 4: gentamicin + SC melatonin (250 µg); Group 5: tobramycin + SC melatonin; Group 6: gentamicin + IM (250 µg) melatonin; Grupo 7: tobramicin + IM melatonina (250 µg); Group 8: gentamicin + DR melatonina; Group 9: tobramycin + DW (10 mg/L) melatonin	5 days – day 1/day 3/day 5 – DPOEA antibiogram	Group 1: control: no significant differences in DPOEA pre and pos treatment. Group 2: gentamicin: Significant differences on DPOEA, pre and post treatment. Group 3: tobramycin: less significant differences comparing to the groups with gentamicin and gentamicin + melatonin. No significant differences between the groups that received melatonin in difference ways of administration. The antibiograms showed that the efficacy of the aminoglycosides was not affected by the use of melatonin.
Lopez-Gonzalez et al.^24^ (2000)	140 Rodents — Wistar Female Rat two months old 20 in each group	7 Groups - Group 1: control; Group 2: solvents and melatonin: Group 3: cisplatin; Group 4: cisplatin + melatonin DW (10 mg/L), Group 5: cisplatin + melatonin SC (250 µ); Group 6: cisplatin + antioxidant mixture; Group 7: cisplatin + melatonin SC + antioxidant.	DPOEA: day 0, day 7, day 15	Day 7 pos-treatment: decrease with values from animals treated with cisplatin and a more moderate decrease in the animals treat with the combined therapies day 15 post-treatment: decrease values in group treated with cisplatin. The groups treated with cisplatin + melatonin or other antioxidant mixture presented values to the group control.
Ye et al.^25^ (2009)	54 Guinea pigs age and sex not mentioned	4 Groups: Group 1 — 13 animals, gentamicin (120 mg/kg/day); Group 2: 13 animals: gentamicin (120 mg/kg/day) + melatonin (0.3 mL/kg/day); Group 3: 11 animals: melatonin (0.3 mL/kg/day); Group 4: 11 animals: saline.	17 days of injection and then was realized DPOAE. Histologic evaluation.	17 days of injection and then was realized DPOAE. Group 3 and 4 melatonin and saline — stable hearing at all of the DPOAE. group of gentamicin showed the expected hearing loss between 3 and 8 kHz. Animals treat with gentamicin + melatonin showed reduced lower hearing loss at all frequencies, compared with gentamicin group, this difference was statistically significant at 3, 4, 6 and 8 kHz. Cochlear histopathology showed melatonin treatment exert a protective effect on the outer cells. The group of gentamicin showed almost complete loss of outer hair cells. Histologic results showed that there was a more significant loss of outers cells in Group 1 than Group 2. Loss of cells in Group 3 was similar to the Group 2.

The included studies obtained a “score” 5,^23^, ^24^, ^25^ “score” 7^19^, ^26^ and “score” 8^17^, ^27^ by CAMARADES, indicating achieving methodological quality, especially in the calculation the sample size, animal model used and the use of otoacoustic emissions to evaluate the results, positive items in all selected studies. Regarding compliance with animal welfare regulations, except study^25^ did not score positive. In Refs. 23, 24, 25 as for blind drug therapy, only Refs. 25, 26, 27 did not obtain positive scores. In the item that evaluates random allocation to treatment or control, the studies^19^, ^23^, ^24^, ^26^^,^^27^ did not obtain a positive score. Blind evaluation of results was performed, except in Refs. 19, 23, 24, 25, 28. The declaration of potential conflict of interest was not presented by^19^, ^23^, ^24^, ^25^ studies. As it can be seen at (Table 2).

**Table 2:** CAMARADES quality of assessment checklist.

Autor, year	(1)	(2)	(3)	(4)	(5)	(6)	(7)	(8)	(9)	(10)	Total
Lopez-Gonzalez et al.,^23^ 2000	N	N	N	Y	N	Y	Y	Y	Y	N	5
Lopez-Gonzalez et al.,^24^ 2000	N	N	N	Y	N	Y	Y	Y	Y	N	5
Erdem et al.,^19^ 2005	Y	Y	N	Y	N	Y	Y	Y	Y	N	7
Ye et al.,^25^ 2009	Y	N	Y	N	N	Y	Y	Y	U	N	5
Bas et al.,^27^ 2012	Y	Y	N	N	Y	Y	Y	Y	Y	Y	8
Demir et al.,^26^ 2015	Y	Y	N	N	Y	Y	Y	Y	Y	Y	7
de Araujo et al.,^17^ 2019	Y	Y	Y	Y	N	Y	Y	Y	Y	Y	9

(1) Peer reviewed publication; (2) control of temperature; (3) random allocation to treatment or control; (4) blinded therapy; (5) blinded assessment of outcome; (6) use of emission otoacustic evocade to evaluate the outcomes.; (7) animal model (ionizing radiation); (8) sample size calculation; (9) compliance with animal welfare regulations; (10) statement of potential conflict of interests.

**Figure 2:**
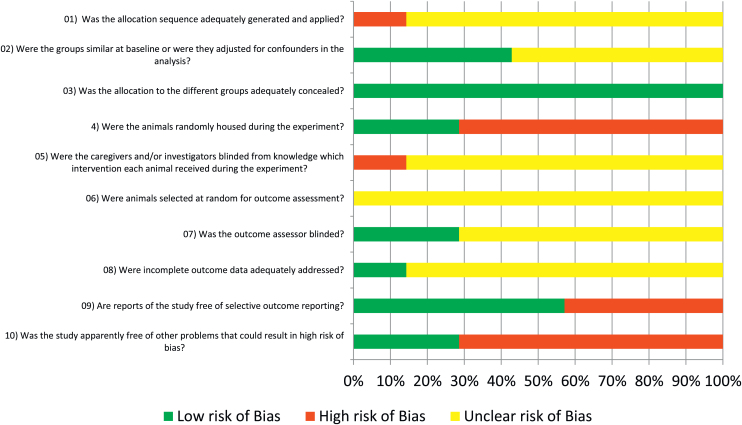
“Syrcle” RoB risk of bias of the included articles.

The protocol for evaluating the risk of bias using the SYRCLE RoB revealed a high risk of bias in all eligible studies, portraying inadequate randomization, unconcealed allocation, blinding of caregivers and evaluators (Fig. 2).

The studies by Erdem et al.,^19^ Ye et al.^25^ and Demir et al.^26^ did not provide sufficient data for inclusion in the meta-analysis in any of the assessed outcomes (OAE, BERA, or histological analysis). Thus, they were excluded and contributed only to the systematic review. The studies that were included in the meta-analysis were works by Lopez-Gonzalez et al.,^23^, ^24^ Bas et al.,^27^ and de Araujo et al.^17^ The study by Bas et al.^27^ was separated into two because it featured independent control and experimental groups.

**Figure 3:**
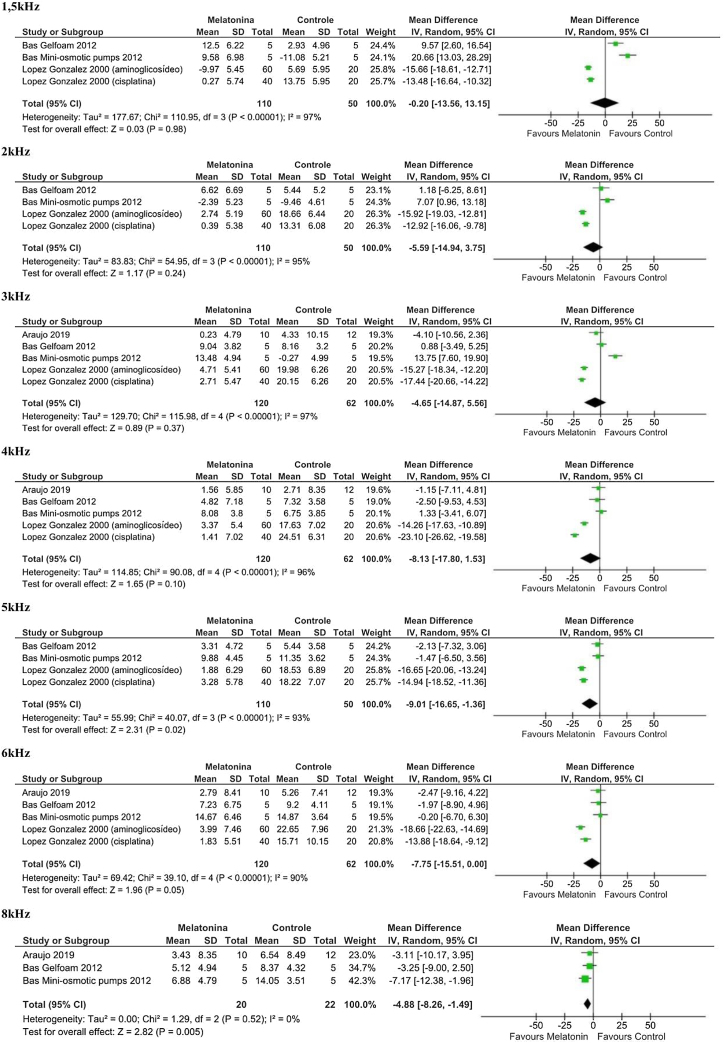
Forest Plot of the analysis of the frequencies from 1500 Hz to 8000 Hz, where we can see less heterogeneity at the frequency of 8000 Hz compared to the other outcomes.

Of the total outcomes that were evaluated, only OAE data were available in the selected studies. We included OAE amplitude values at 1500, 2000, 3000, 4000, 5000, 6000, and 8000 Hz (Fig. 3), with a minimum of three studies for each frequency evaluated.

At 5000 Hz, 6000 Hz, and 8000 Hz frequencies, there was a protective effect of melatonin in preventing the reduction in OAEP amplitude observed during ototoxic drug use (5000 Hz *p* < 0.02; 6000 Hz *p* < 0.05; 8000 Hz *p* < 0.005). At 5000 Hz the difference in means was −9.01 (95% CI −16.65 to −1.36; *p* = 0.02; I^2^ = 93%). At 6000 Hz the difference in means was −7.75 (95% CI −15.51 to 0.00; *p* = 0.05; I^2^ = 90%). At 8000 Hz the difference in means was −4.88 (95% CI −8.26 to −1.49; *p* = 0.005; I^2^ = 0%).

At more severe frequencies (1500 Hz, 2000 Hz, 3000 Hz, and 4000 Hz), no protective effect of melatonin was demonstrated, although the meta-analysis showed a trend towards this effect. At these frequencies, the confidence interval encompassed the value 0, not allowing conclusions to be drawn. At 1500 Hz, the difference in means was −0.20 (95% CI −13.56 to 13.15; *p* = 0.98; I^2^ = 97%). At 2000 Hz, the difference in means was −5.59 (95% CI −14.94 to 3.75; *p* = 0.24; I = 95%). At 3000 Hz, the difference in means was −4.65 (95% CI −14.87 to 5.56; *p* = 0.37; I^2^ = 97%). At 4000 Hz, the difference in means was −8.13; 95% CI −17.80 to 1.53; *p* = 0.10; I^2^ = 96%.

## Discussion

In the frequencies 5000 Hz, 6000 Hz, and 8000 Hz, we noticed a minor reduction in OAE amplitude values after the administration of ototoxic drugs in the groups that used melatonin. This was noticed even when the heterogeneity was high in the frequencies of 5000 Hz and 6000 Hz. At more severe frequencies (1500 Hz, 2000 Hz, 3000 Hz, and 4000 Hz), no protective effect of melatonin associated with ototoxic drugs was evidenced. This is the first known systematic review that evaluate the otoprotective effect of melatonin in a series of experimental studies and shows a potential effect against the toxic action of antibiotics and chemotherapeutics on the hair cells of the cochlea. This would provide relevant information for human translational interventions in the future.

The studies by Lopez-Gonzalez et al.,^23^ Ye et al.,^25^ and Bas et al.,^27^ used gentamicin and tobramycin as ototoxic agents. Lopez-Gonzalez et al.^23^ tested the efficacy of melatonin administered by three routes: intramuscular (250 µg), subcutaneous (250 µg), and oral (10 mg/L). The study investigated the association of the route of administration with two different aminoglycosides: gentamicin (160 mg/kg by weight) and tobramycin (200 mg/kg by weight) for 5 days. The melatonin administration started one week before the application of the aminoglycosides and ended on the last day of the experiment. This study showed otoprotection in the groups that used gentamicin associated with melatonin, regardless of the route of administration. When evaluated in association with tobramycin, the evaluation of melatonin as an otologic protective agent did not show otoprotection.

Ye et al.^25^ used gentamicin (120 mg/kg/day) associated with melatonin (0.3 mL/kg/day). The outcomes were favorable to otoprotection in the groups that used melatonin, demonstrating reduced hearing loss in the results of OAE evaluation and cochlear histopathology.

Bas et al.^27^ used gentamicin in a single dose (10 mg) that was administered by local routes (micro-osmotic pump and geofoam) with concentrations of 500 µg/200 µL of saline solution in the first and 500 µg/40 µL of saline solution in the second. Melatonin was also administered in a single dose, at a dose of 10 mg. The results obtained were similar to the study by Lopez-Gonzalez et al.,^23^ confirming the otoprotective efficacy of melatonin against the ototoxic effect of gentamicin as seen in both OAE and BAEP results. This result was regardless of the administration protocol.

Among the studies that evaluated the effect of melatonin on the ototoxicity of antibiotics, the study by Erdem et al.^19^ was the only one that used amikacin (600 mg/kg, intramuscularly). The objective was to test which dose of melatonin would be effective in otoprotection: low (0.4 mg/kg) or high (4.0 mg/kg), upon daily intraperitoneal administration. Through the evaluation of OAE, we concluded that while low-dose melatonin protected the inner ear from ototoxicity, high-dose supply potentiated the effects of amikacin-induced ototoxicity. This can be attributed to vasodilatory effect of melatonin that contributes to the accumulation of the drug in the inner ear. There is no data in the study to infer the ideal dose of melatonin, and further studies are needed a comprehensive understanding of the same.

de Araujo et al.,^17^ Demir et al.^26^ and Lopez-Gonzalez et al.^24^ used cisplatin as an ototoxic agent. In the study by de Araujo et al.,^17^ 10 mg/kg of the single dose cisplatin was administered intraperitoneally in the control and experimental groups. The dose of melatonin used in the experimental group was 1 mg/kg/day, also intraperitoneally.

Demir et al.^26^ used 12 mg/kg of single-dose cisplatin in the control and experimental groups for five days. Intratympanic administration of melatonin (0.1 mg/mL) was carried out 30 min before the application of cisplatin in the experimental group.

Lopez-Gonzalez et al.^24^ administered cisplatin (10 mg/kg; intraperitoneally; single dose) in two groups: 1) melatonin orally at a dose of 1 µmg/mL dissolved in water, and 2) melatonin (250 µg) subcutaneously. The administration of melatonin was started seven days before the administration of cisplatin and ended at the time of experiment conclusion. The outcomes of the three studies were favorable to the otoprotective effect of melatonin to reduce the decrease in otoacoustic emission amplitude in the groups. This was evident as per the OAE examinations performed in the three studies and histological analysis performed in the study by Demir et al.^26^

Despite the positive outcomes described in the selected studies, a positive effect of using melatonin against ototoxicity was observed only at frequencies of 5000 Hz, 6000 Hz, and 8000 Hz. In contrast, the protective effect of melatonin was not demonstrated at more severe frequencies (1500 Hz, 2000 Hz, 3000 Hz, and 4000 Hz).

However, there is a trend towards a beneficial effect of melatonin at these frequencies. Thus, the 95% Confidence Interval encompassed the value zero, thereby disallowing the verification of the protective effect of melatonin.

The cellular changes involved in the ototoxicity of aminoglycosides and cisplatin are known to begin in the outer hair cell layer of the basal gyrus of the cochlea. This is considered to be the most susceptible region to ototoxic effects.^29^ With continued exposure, the damage extends to the apex of the cochlea, encompassing the inner hair cells. With progression, other cells may be affected, such as sustentation cells, vascular stria cells, and even nerve cells of the spiral ganglion.^29^, ^30^

As the cellular damage starts in the basal gyrus of the cochlea, the audiological changes are first identified at the higher frequencies. This fact may justify that, when analyzing the higher frequencies, the magnitude of the effect of melatonin use is more important and thus more easily identified in the studies. Thus, in future studies, the analysis of acute frequencies is necessary to establish the real role of melatonin in imparting necessary protection against ototoxic agents.

The initial damage to the basal regions of the cochlea is also observed in cases of hearing loss due to the aging process. The study by Serra et al.^20^ showed that in an animal model of rodents with aging hearing loss, the effect of melatonin is more critical in the cochlear regions of high frequencies. Considering that the process of cell death is similar to that seen in cases of ototoxicity, it is expected that the results are more evident in the high frequencies and better visualized in this cochlear region.

Methodological analysis of the studies mentioned above highlights an important point about the evaluations of de Araujo et al.^17^ and Ye et al.^25^ These were in the only studies wherein animals were randomly distributed, and the outcomes were evaluated blindly by the authors, including the associated statistical analysis. This data becomes relevant for the outcomes analyzed by OAEs since the variability of results depends on the execution of the exam.

It can be stated that although the studies are consistent and innovative, there are methodological gaps that prevent their replicability and reliable comparative evaluation. Some aspects weaken the comparison between the investigations, thereby limiting the possibility of consistent conclusions.

The main limitation of this meta-analysis study was the heterogeneity observed in evaluating some frequencies. This fact may be explained by the existence of few studies on the subject. Most studies evaluated a small number of animals, and little knowledge is available about the curve “dose × effect of melatonin”, on the use of various routes of administration, different ototoxic drugs, without the signaling of a protocol considered “gold standard” in animal models for these tests.

In addition, the dearth of studies conducted at higher frequencies (above 8000 Hz) is considered an important limitation in the field. This high frequency is generally the range in which ototoxicity is known to occur. Thus, it is possible to assume that data evaluating higher frequencies may present results with less heterogeneity, thereby making the meta-analysis even more robust and conclusive.

## Conclusion

This systematic review showed, by preclinical evidences, that melatonin could provide protection against the ototoxic effects of cisplatin and aminoglycosides at the frequencies 5000 Hz, 6000 Hz, and 8000 Hz. The same effect was not observed in the lower frequencies. The methodology of the available papers did not meet the necessary methodological rigor that promotes the safe replicability of these studies. New experimental studies that seek to establish a dose-effect curve of melatonin, the gold standard route for its administration and preparation, should be encouraged to define its real role in otoprotection for future translation of the results to prevent human ototoxicity.

## Authors’ contributions

NLA participated in the study design, data collection, analysis, discussion, writing and critical review of the manuscript; LSMS participated in the study design, analysis, discussion, writing and critical review of the manuscript; JGA participated in the discussion, writing and critical review of the manuscript; VVR participated in the study design, analysis, discussion and, writing critical review of the manuscript; MLQS and AAC participated in the analysis and critical review of the manuscript; ALLS participated in the study design, analysis, discussion, writing and critical review of the manuscript.

## Conflicts of interest

The authors declare no conflicts of interest.
